# SUMO and SUMO-Conjugating Enzyme E2 UBC9 Are Involved in White Spot Syndrome Virus Infection in *Fenneropenaeus chinensis*

**DOI:** 10.1371/journal.pone.0150324

**Published:** 2016-02-29

**Authors:** Xiaoqian Tang, Wei Li, Jing Xing, Xiuzhen Sheng, Wenbin Zhan

**Affiliations:** 1 Laboratory of Pathology and Immunology of Aquatic Animals, Ocean University of China, Yushan road 5, Qingdao, 266003, PR China; 2 Laboratory for Marine Fisheries Science and Food Production Processes, Qingdao National Laboratory for Marine Science and Technology, No. 1 Wenhai Road, Aoshanwei Town, Jimo, Qingdao, 266071, China; Shanghai Ocean University, CHINA

## Abstract

In previous work, small ubiquitin-like modifier (SUMO) in hemocytes of Chinese shrimp *Fenneropenaeus chinensis* was found to be up-regulated post-white spot syndrome virus (WSSV) infection using proteomic approach. However, the role of SUMO in viral infection is still unclear. In the present work, full length cDNAs of SUMO (*FcSUMO*) and SUMO-conjugating enzyme E2 UBC9 (*FcUBC9*) were cloned from *F*. *chinensis* using rapid amplification of cDNA ends approach. The open reading frame (ORF) of *FcSUMO* encoded a 93 amino acids peptide with the predicted molecular weight (M.W) of 10.55 kDa, and the UBC9 ORF encoded a 160 amino acids peptide with the predicted M.W of 18.35 kDa. By quantitative real-time RT-PCR, higher mRNA transcription levels of *FcSUMO* and *FcUBC9* were detected in hemocytes and ovary of *F*. *chinensis*, and the two genes were significantly up-regulated post WSSV infection. Subsequently, the recombinant proteins of *FcSUMO* and *FcUBC9* were expressed in *Escherichia coli* BL21 (DE3), and employed as immunogens for the production of polyclonal antibody (PAb). Indirect immunofluorescence assay revealed that the FcSUMO and UBC9 proteins were mainly located in the hemocytes nuclei. By western blotting, a 13.5 kDa protein and a 18.7 kDa protein in hemocytes were recognized by the PAb against SUMO or UBC9 respectively. Furthermore, gene silencing of *FcSUMO* and *FcUBC9* were performed using RNA interference, and the results showed that the number of WSSV copies and the viral gene expressions were inhibited by knockdown of either SUMO or UBC9, and the mortalities of shrimp were also reduced. These results indicated that *FcSUMO* and *FcUBC9* played important roles in WSSV infection.

## Introduction

Small ubiquitin-like modifiers (SUMO) are a family of small proteins that could covalently attach to and detached from other proteins in cells to modify their functions. The process of covalent and reversible attaching of SUMO moiety to a target protein was known as SUMOylation, which was an important post-translational modification and involved in various cellular processes [1–3]. Although amino acid sequence of SUMO is similarly to ubiquitin, SUMOylation does not typically lead to degradation of the substrate and instead has a more diverse array of effects on substrate function, such as nuclear-cytosolic transport, transcriptional regulation, apoptosis, protein stability, response to stress and antiviral defense [4,5]. In mammalian cells, four SUMO family members were identified, namely, SUMO-1, -2, -3 and -4, whereas in invertebrates there is only a single SUMO gene [6,7]. The conjugation of SUMO to target proteins involves three classes of enzymes, E1 activating enzyme, E2 conjugating enzyme and E3 target specificity enzyme [8], and the Ubc9 is the unique SUMO E2 enzyme known to conjugate SUMO to target substrates [9–10]. The Ubc9 serves as a lynchpin in the SUMO conjugation pathway, interacting with the SUMO E1 during activation, with thioester linked SUMO after E1 transfer and with the substrate and SUMO E3 ligases during conjugation [11].

White spot syndrome virus (WSSV) is one of the most devastating viral pathogens in shrimp, and caused considerable economic losses to shrimp culture industry worldwide [12]. In our previous research, SUMO in hemocytes of Chinese shrimp *Fenneropenaeus chinensis* was found to be significantly up-regulated at both mRNA and protein levels post WSSV infection [13]. And a recent research demonstrated that WSSV Immediate early (*ie*) proteins could be modified by crayfish SUMOylation, and the modification would benefit WSSV replication [1]. All these results implied that SUMO and UBC9 played important roles in WSSV infection. Up to now, the SUMO and UBC9 cDNAs have been cloned in some crustaceans, including *Litopenaeus vannamei* [14], *Procambarus clarkia* [1], *Eriocheir sinensis* [15] and *Scylla paramamosain* [16]. However, the knowledge on SUMO and UBC9 of shrimp in viral infection is still limited.

In the present work, full length cDNAs of SUMO (*FcSUMO*) and UBC9 (*FcUBC9*) in *F*. *chinensis* were cloned and characterized, and their distribution characteristics were both determined at gene and protein levels. Moreover, the potential roles of SUMO and UBC9 in WSSV infection were further investigated *in vivo* by RNA interference (RNAi).

## Materials and Methods

### Shrimp and sample preparation

Ministry of Agriculture of China allows the Chinese shrimp to be caught from Yellow Sea of China before and after fishing-moratorium period, and the shrimps used in the present study were caught after the fishing-moratorium period. The grossly healthy Chinese shrimps with average size of 15–17 cm in length were caught from Yellow Sea of China, which were all negative for WSSV by PCR assay according to previously described method [17]. Eight tissues, including hemocytes, lymphoid organ, ovary, heart, intestine, muscle, gill and hepatopancreas were sampled from 12 healthy shrimps. For WSSV challenge experiment, shrimps were acclimatized for 5 days at 25°C. Each shrimp was intra-muscularly injected with 100 μl WSSV inoculum (10^7^ copies) prepared according to the previous method [13]. Shrimps were injected with 100 μl phosphate-buffered saline (PBS, pH 7.4) as control. The hemocytes and ovary were sampled from 6 randomly selected shrimps in each group before infection and at 6, 12, 24, 36, 48, 60 and 72 h post infection (hpi) as previously described [18].

### Cloning and sequencing of *FcSUMO* and *FcUBC9* cDNA

The partical cDNA fragments of SUMO and UBC9 were amplified by RT-PCR from shrimp hemocytes RNA using their respective degenerate primers, which were designed based on the conserved region of other known SUMO or UBC9 sequences. The PCR amplification and the purification, cloning and sequencing of PCR products were performed according to the previous method [19].

To obtain the full-length cDNA sequences, gene specific primers of SUMO and UBC9 were designed respectively based on their partial cDNA sequences obtained, and rapid amplifications of cDNA ends (RACE) were performed using the SMART RACE cDNA Amplification Kit (Clontech, USA) according to the manufacturer’s instruction. The RACE products were purified, cloned, and sequenced. The full-length cDNAs of *FcSUMO* and *FcUBC9* were obtained by ligation of their overlapping cDNA fragments. All of the primers used in the present work were listed in Table 1.

**Table 1:** Primers used in this work.

Primer	Primer sequence (5’-3’)
Degenerate primer	
SUMO1-F	AYAATGTCYGATAACRCTGACGC
SUMO1-R	GTYTGYTCCTGGTAVACYTCAAT
UBC91-F	TGGAGRAARGAYCAYCCNTTTG
UBC91-R	TTACTCRAADGGDGCHGACATKGC
5’ and 3’ RACE cloning	
SUMO-3’	GGAAATGGAGAATGATGACG
UBC9-3’	CCTTTTGGGTATTCAGGACTTGC
SUMO-5’	CATCTGTGTGGTCATCTTCACTCGG
UBC9-5’	GTCGCCAGTCCTTCTCTTCATCCAG
Recombinant expression	
rSUMO-F	CGGAATTCATGTCTGATAACGCTGACGC
rSUMO-R	CCCAAGCTTATGGCCGCCGGTCTGCT
rUBC9-F	CGGAATTCATGTCTGGAATTGCCATTG
rUBC9-R	CCCAAGCTTCTCAAATGGAGCGGACAT
RNAi	
SUMOi-F	TAATACGACTCACTATAGGGGAAGGGGAAGGGAACGAATA
SUMOi-R	TAATACGACTCACTATAGGGTGGTCTGTGCGAAAATCTCA
UBC9i-F	TAATACGACTCACTATAGGGGAATTGCCATTGCACGACTA
UBC9i-R	TAATACGACTCACTATAGGGGCCTGGGCTCGTACTCTTTT
GFPi-F	TAATACGACTCACTATAGGGGTGCCCATCCTGGTCGAGCT
GFPi-R	TAATACGACTCACTATAGGGTGCACGCTGCCGTCCTCGAT
Quantitative and semi-quantitative RT-PCR	
SUMO2-F	CAGAAGGGGAAGGGAACGA
SUMO2-R	AACGCAGCGATGCTACAGG
UBC92-F	CTCTGTTCCACCCCAATGTT
UBC92-R	AGCAAGTCCTGAATACCCAAA
18S-F	ACAATGGCTATCACGGGTAACG
18S-R	CTGCTGCCTTCCTTAGATGTGGTA
*wsv*051-F	CACTTGCGGCTACACCTTT
*wsv*051-R	CTTGGGACCTCTGTGGATTT
*ie*1-F	CAACAACAGACCCTACCCG
*ie*1-R	AATACGACATAGCACCTCCACT
*ie*2-F	CATCATTCCCTCGTCATCG
*ie*2-R	ACTTCCTGGGCAATCCTCT
*wsv*477-F	TGGAAGAAAGAGCAAGGAAAAG
*wsv*477-R	CAAAAGGGCACAACAAATCAC
*dnapol*-F	CATTTATGAGCGGGGACG
*danpol*-R	TGGGTAATATCAGCCAGCAT
*vp*28-F	AAACCTCCGCATTCCTGTGA
*vp*28-R	TCCGCATCTTCTTCCTTCAT
*vp*26-F	TTGGGACTCGCATCATCTG
*vp*26- R	TTGGCAACCTAACAAACCTG
*vp*24-F	TTGCCAGGAGAAAATCGC
*vp*24- R	CTTTACTTGGAGACGGAGACC
*vp*19-F	GCGGAAACGAGGAACAGA
*vp*19- R	GGATGGGACCAAAGAAGGA
*vp*15-F	ATGACAAAATACCCCGAGAAC
*vp*15- R	GGAGATGCGTCCAGCAGT

### Sequence analysis

The homology search of nucleotide and protein sequences of *FcSUMO* and *FcUBC9* was conducted with BLAST (http://www.ncbi.nlm.gov/blast/). The deduced amino acid sequences and predicted protein motifs were generated using ExPASy tools (http://www.us.expasy.org/) and SMART (http://smart.embl-heidelberg.de/). Multiple alignment was generated with the ClustalW multiple alignment programs (http://www.ebi.ac.uk/Tools/msa/clustalw2), and MEGA 4.0 was used to construct phylogenetic trees [20].

### Tissue distribution of FcSUMO and FcUBC9 mRNA

Total RNA was extracted from different tissues of shrimp using TRIzol reagent (Invitrogen, USA), and then treated with RNase-free DNase I (TaKaRa, Japan). The first-strand cDNA was synthesized from 2 μg of DNA-free total RNA by M-MLV reverse transcriptase (Promega, USA) according to the manufacturer’s protocol. Quantitative real-time RT-PCR (qRT-PCR) was used to analyze the mRNA expression levels of *Fc*SUMO and *Fc*UBC9 in different tissues. The gene specific primers SUMO-F4 and SUMO-R4 were used to amplify a 150 bp product of *FcSUMO*, and specific primer pairs UBC9-F4 and UBC9-R4 were used to amplify a 121 bp product of *FcUBC9*, while the 18S rRNA primer pair 18S-F and 18S-R were used for amplification of the internal control fragment for qRT-PCR. PCR was carried out using SYBR Premix Ex Taq^™^ (Takara) in a Thermal Cycler Dice^®^ Real Time System (Eppendorf, Germany) with the following conditions: 95°C for 2 min, followed by 40 cycles of 95°C for 10 s, 58°C for 10 s, and 72°C for 20 s. A dissociation curve with a single peak was used to monitoring the amplified product. The data were calculated according to 2^-ΔΔCt^ method.

### Detection of *FcSUMO* and *FcUBC9* mRNA expressions post WSSV infection

Total RNA of hemocytes and ovary were prepared from the WSSV infected shrimps sampled at various time points as above, and qRT-PCR was performed to investigate the effects of WSSV infection on *FcSUMO* and *FcUBC9* transcript levels respectively. The qRT-PCR was carried out according to the methods described above.

### Recombinant expression and purification of *FcSUMO* and *FcUBC9*

The *FcSUMO* and *FcUBC9* open reading frame (ORF) genes were amplified by PCR using specific primer pairs rSUMO-F/rSUMO-R and rUBC9-F/rUBC9-R respectively. After confirmation by sequencing, the purified PCR products were cloned into pET-28a vector to obtain the recombinant plasmids (pET-28a-SUMO and pET-28a-UBC9), then transformed into *Escherichia coli* BL21 (DE3) (Novagen). Positive clones were screened by PCR and confirmed by sequencing, then incubated in LB medium and induced with isopropyl-β-D-thiogalactosidase (IPTG). The recombinant *FcSUMO* (rSUMO) and *FcUBC9* (rUBC9) with 6×His-tag were purified with Ni^2+^-afinity column (HiTrap HP column, GE) respectively. Subsequently, the purified protein was renatured in TBS glycerol buffer (50 mM Tris-HCl, 50 mM NaCl, 10% glycerol, 6–0 M urea, pH 8.0) by four dialysis steps and each dialysis step was performed at 4°C for 12 h. Finally, the proteins were analyzed by SDS-PAGE and stained with Coomassie brilliant blue R250. The concentration of the two purified proteins was quantified by bicinchoninic acid (BCA) method [21].

### Production of rSUMO and rUBC9 antisera

Purified rSUMO and rUBC9 fusion protein were used to immunize BALB/c mice to obtain the antisera respectively. The immunization procedure was performed as previously described [22]. The reactivities of the PAbs were determined by western blotting. Briefly, the purified rSUMO and rUBC9 were separated by SDS-PAGE and then transferred onto PVDF membrane (Millipore, USA). After blocking with 4% bovine serum albumin (BSA) in PBS for 1 h at 37°C, the membrane was incubated with PAb against rSUMO or rUBC9 for 1 h at 37°C. After washing thrice with PBST (PBS containing 0.05% Tween 20), goat-anti-mouse Ig-alkaline phosphatase antibody (1:4000, Sigma) was added for 1 h incubation at 37°C. Positive bands were developed with substrate solution (100 mM NaCl, 100 mM Tris and 5 mM MgCl_2_, pH 9.5) containing 5-bromo-4-chloro-3-indolyphosphate (BCIP, Sigma) and nitroblue tetrazolium (NBT, Sigma) for 20 min, and stopped by washing with distilled water. The PAbs were replaced by sera of unimmunized mice as control.

### Characterization of SUMO and UBC9 in hemocytes of *F*. *chinensis* by Western blotting and indirect immunofluorescence assay (IIFA)

For western blotting, collected hemocytes were lysed in Western and IP buffer (Beyotime, China), and then the cell lysate was centrifuged at 4°C for 20 min at 13,000 rpm. The supernatant was collected and subjected to SDS-PAGE. And then the samples were transferred onto PVDF membrane and subjected to the procedures described above for detection of SUMO and UBC9 in hemocytes of *F*.*chinensis*.

For IIFA, the hemocytes were suspended in PHPBS (377 mM NaCl, 2.70 mM KCl, 8.09 mM Na_2_HPO_4_, 1.47 mM K_2_PO_4_, pH 7.4, 780 mOsm·L), settled onto glass sliders for 30 min subsiding, and then fixed with acetone for 15 min. The slides were overlaid with PAb against rSUMO or rUBC9. After incubation for 1 h at 37°C in a moist chamber, the sliders were rinsed thrice with PHPBS for 5 min each time and incubated with goat-anti-mouse Ig-FITC (1:256, Sigma), contained Evan’s blue dye (EBD) as the counterstain, for 1 h at 37°C in the dark, and DAPI staining (blue) was used to visualize cell nuclei. Finally, the slides were rinsed again and observed by fluorescence microscope. The PAbs were replaced by sera of unimmunized mice as control.

### RNA interference and WSSV infection

Double-stranded RNA (dsRNA) corresponding to *FcSUMO*, *FcUBC9* and green fluorescent protein gene (*GFP*) sequences were generated by *in vitro* transcription. DNA templates for dsRNA preparation were amplified by PCR using specific primers, SUMOi-F and SUMOi-R for *FcSUMO*, UBC9i-F and UBC9i-R for *FcUBC9*, EGFPi-F and EGFPi-R for *GFP* (Table 1), which were designed with E-RNA*i* (http://www.dkfz.de/signaling/e-rnai3/idseq.php). The PCR products were purified, and 1 mg of each template was used in an *in vitro* transcription reaction (MBI Fermentas, USA) according to the manufacturer’s protocol. The sense and anti-sense single stranded RNA were then mixed at equimolar amounts and annealed to construct the dsRNAs. The quality of dsRNAs was verified by agarose gel electrophoresis, and the dsRNAs were quantified by NanoDrop spectrophotometer (Thermo Scientific, USA). Then the concentration of dsRNAs was adjusted to 600 μg·ml^-1^. Shrimps were divided into four groups, SUMOi group, UBC9i group, negative control group and blank control group, and injected with 100 μl dsSUMO, dsUBC9, dsGFP and TNE buffer respectively. qRT-PCR was carried out to confirm the effect of target gene interference within 5 days at 12-h intervals.

To investigate the effects of suppression of the *FcSUMO* and *FcUBC9* transcripts on WSSV infection, shrimp were grouped and injected with dsRNA or TNE buffer as described above, and at 48 h after the injection, shrimp were injected with 100 μl WSSV inoculum (10^7^ copies), and shrimp injected with equal volume of PBS was served as blank control group. Each treatment was replicated with three batches of 50 shrimp. Total hemocyte genomic DNA was extracted using DNA extraction kit (Takara) before WSSV infection and at 6, 12, 24, 36, 48, 72 h post infection. An equal quantity of DNA (50 ng) was added into the SYBR Green Premix with the WSSV primer set (VF and VR) for quantitative real-time PCR (qPCR). The number of WSSV copies in hemocytes of *F*. *chinensis* at various time points was detected according to our previous work [18]. Furthermore, the expression levels of 10 genes of WSSV, including 3 *ie* genes (*wsv051*, *ie1*and *ie2*), 2 early genes (*wsv477* and *dnapol*) and 5 late genes (*vp28*, *vp26*, *vp24*, *vp19* and *vp15*) at 48 hpi were measured by semi-quantitative RT-PCR. Mortality of each experimental group was recorded daily.

### Statistics

Data were given as arithmetic mean values. The statistical analysis was performed using the software SPSS 19.0. One-way ANOVA and Duncan’s multiple comparisons of the means were done to compare data obtained. Differences were considered significant at *P*< 0.05.

## Results

### Characters and phylogenetic analysisof *FcSUMO* and *FcUBC9*

**Fig 1:**
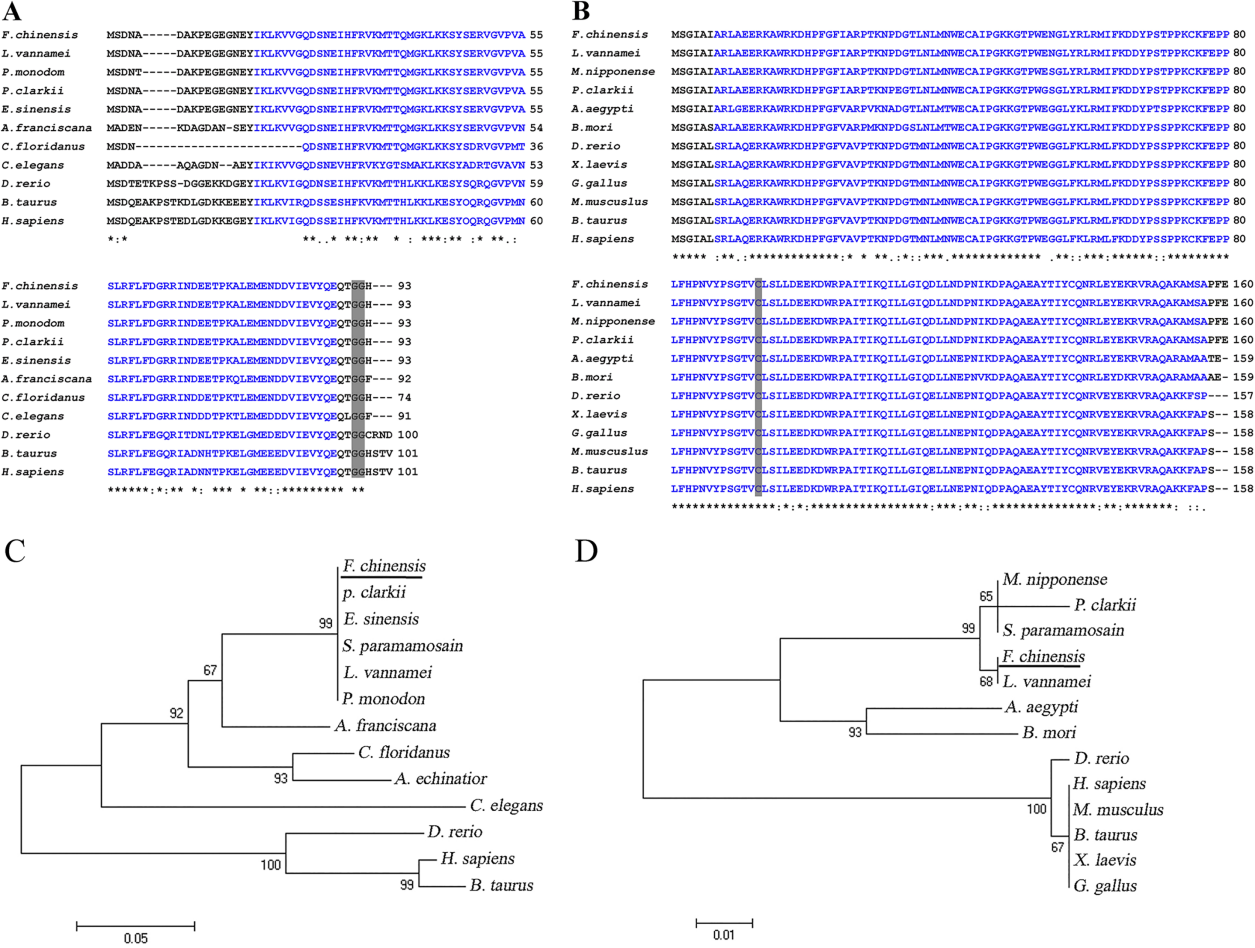
Homology and phylogenetic analysis of *FcSUMO* and *FcUBC9*. Residues in the UBQ domain of FcSUMO (A) and in the UBCc domain of FcUBC9 (B) were in blue, and the active sites (double Gly in SUMO and Cys93 in UBC9) were shaded in gray. SUMO and UBC9 of *F*. *chinensis* were labeled with black underlines in phylogenetic trees of SUMOs (C) and UBC9s (D).

Full length *FcSUMO* cDNA is 1128 bp with an ORF of 282 bp, 5’-untranslated region (UTR) of 65 bp and 3’UTR of 781 bp (GeneBank under accession no. KF773849). The deduced protein contains 93 amino acids with theoretical molecular weight (MW) of 10.55 kDa and isoelectric point (p*Is*) of 4.99 (S1 Fig). Blast homology analysis showed that the deduced amino acid sequence of *FcSUMO* is highly conserved among different species, especially the UBQ domain and the C-terminal double Gly motif (Fig 1A). Moreover, *FcSUMO* was fairly close to SUMOs of other four Decapoda crustaceans, *L*. *vannamei*, *E*. *sinensis*, *P*. *clarkia* and *Penaeus monodon* (99–100% identities). The phylogenetic relationships between *FcSUMO* and other SUMOs were also analyzed by neighbor-joining tree using full amino acid sequences. As shown in Fig 1C, SUMOs of Decapoda crustacean, artemia and nematode formed one major cluster, and SUMOs of fish and mammalian formed another cluster.

Full length UBC9 cDNA is 1170 bp with an ORF of 483 bp, 5’ UTR of 151 bp and 3’UTR of 536 bp (GeneBank accession no. KF773850). The deduced protein contains 160 amino acids with theoretical MW of 18.35 kDa and p*Is* of 8.64 (S1 Fig). Multiple sequences alignment and phylogenetic analysis of UBC9 proteins was also performed. UBC9 shared high similarity with other species, and all UBC9 sequences contained conserved Cys93 residue which is indispensable for binding SUMO (Fig 1B). The sequence identity between *FcUBC9* and three other Decapoda crustaceans, *L*. *vannamei*, *Macrobrachium nipponense* and *P*. *clarkia* was 98–100%. In the phylogenetic tree, the UBC9 of arthropod species grouped together, and UBC9 of vertebrate clustered together (Fig 1D).

### Tissue distribution of *FcSUMO* and *FcUBC9* mRNA

**Fig 2:**
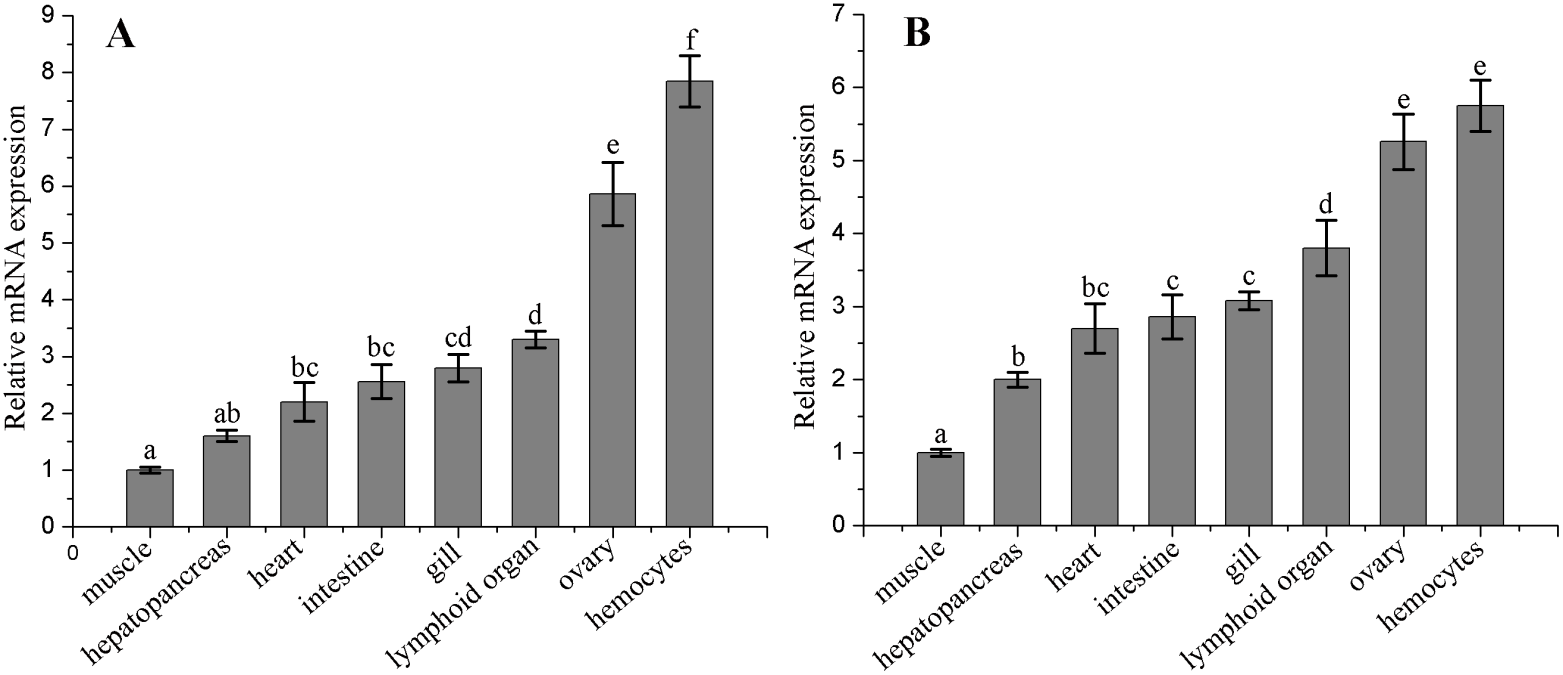
Quantitative real-time RT-PCR analysis of tissue distribution of *FcSUMO* and *FcUBC9* transcripts in healthy *F*. *chinensis*. 18S rRNA served as an internal control. Transcription level of the genes in muscle was normalized to 1. The bar graphs represent the relative fold changes of each tissue, together with error bars which represent mean ± standard deviation (n = 3). *Different letters* indicates significant difference between groups (*p*<0.05). (A) SUMO, (B) UBC9.

The qRT-PCR was employed to detect the *FcSUMO* and *FcUBC9* mRNA expressions in different tissues of healthy shrimp. The *FcSUMO* and *FcUBC9* mRNA expression profiles were highly similar, and the highest transcription level was detected in hemocytes, and high expression levels of *FcSUMO* and *FcUBC9* were also detected in ovary, whereas low expression levels were in muscle and hepatopancreas (Fig 2).

### Expression kinetics of *FcSUMO* and *FcUBC9* post WSSV challenge

**Fig 3:**
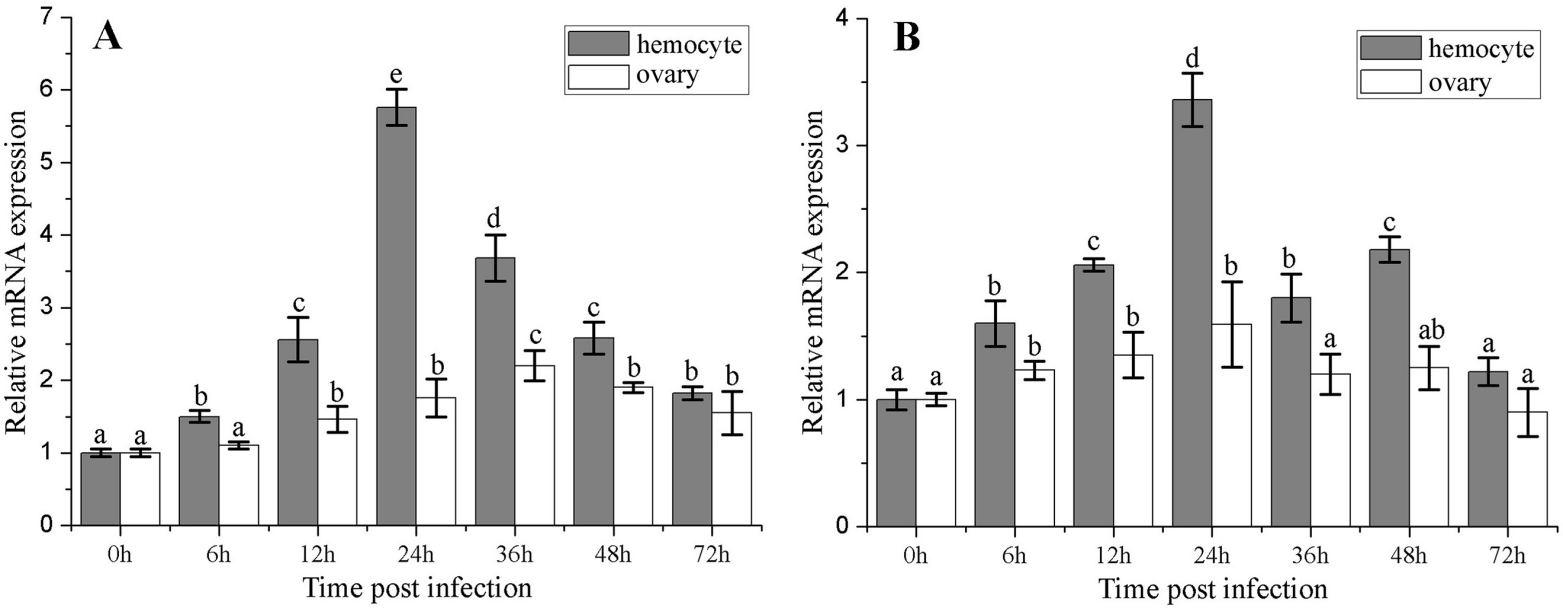
Expression profiles of *FcSUMO* and *FcUBC9* in hemocytes and ovary of *F*. *chinensis* post WSSV infection. 18S rRNA served as an internal control. The bar graphs represent the relative fold changes at each time point infection. The error bars and *Different letters* were used as those in Fig 2. (A) SUMO, (B) UBC9.

The time course of *FcSUMO* and *FcUBC9* expressions in hemocytes and ovary post WSSV infection were investigated by qRT-PCR. The results showed that *FcSUMO* mRNA expression levels in hemocytes and ovary were significantly up-regulated post WSSV infection, and reached the highest level at 24 and 36 hpi respectively (Fig 3A). The *FcUBC9* mRNA expression levels in hemocytes and ovary were also significantly up-regulated post infection, and reached their peak levels at 24 hpi, and then decreased to the control level at 72 hpi (Fig 3B). To be noted, the up-regulation extent of these two genes in hemocytes was much higher that in ovary (Fig 3).

### Characterization of SUMO and UBC9 in hemocytes of *F*. *chinensis*

**Fig 4:**
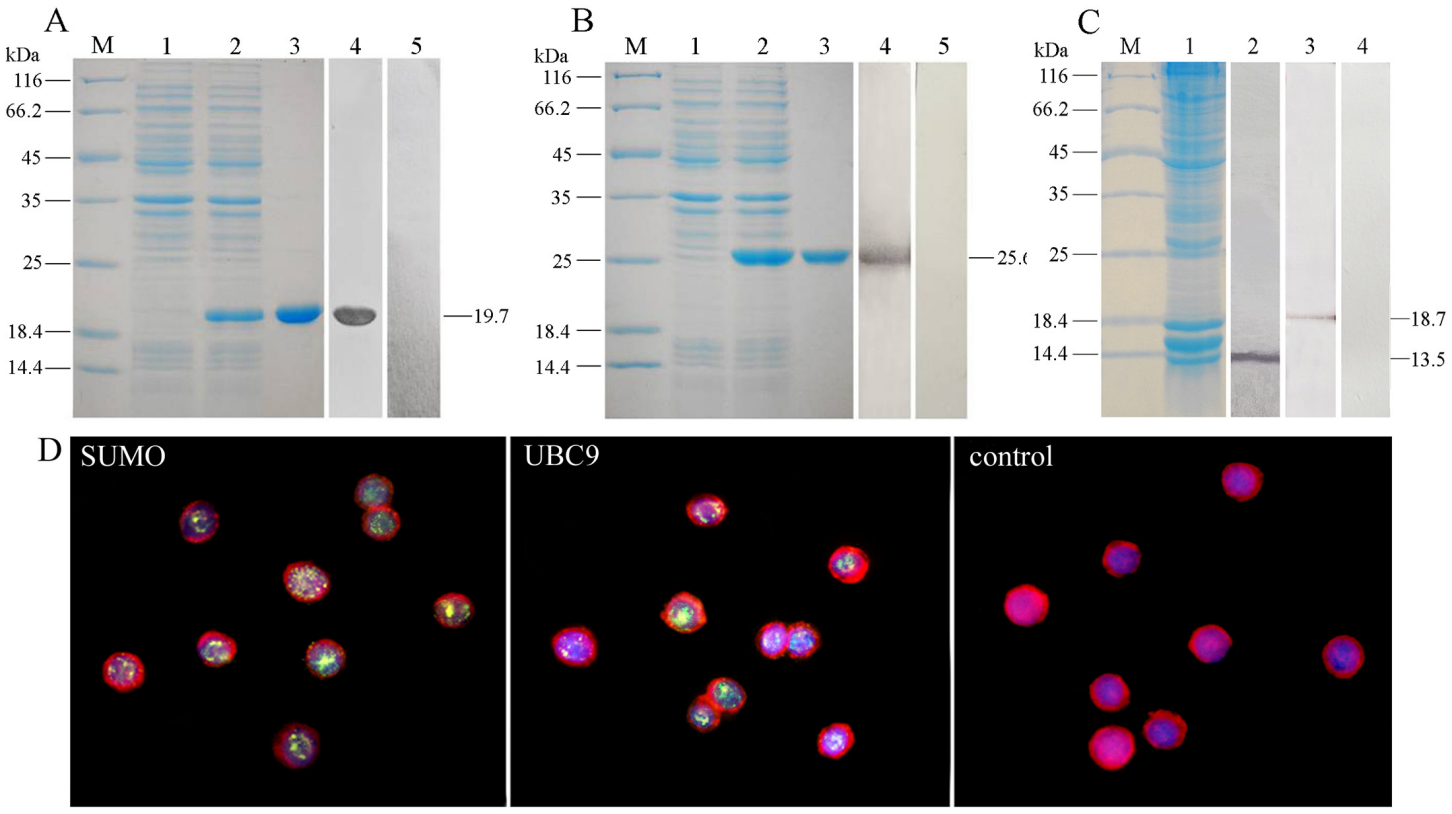
Characterization of SUMO and UBC9 in hemocytes of *F*. *chinensis* by western blotting and IIFA. (A, B) Characterization of PAbs against rSUMO (A) and rUBC9 (B) by western blotting; (C) Characterization of SUMO and UBC9 in hemocytes of *F*. *chinensis* by western blotting; (D) Characterization of SUMO and UBC9 in hemocytes of *F*. *chinensis* by IIFA.

SDS-PAGE revealed that the SUMO and UBC9 with His-tag were successfully expressed in *E*.*coli* BL21 (DE3) with expected MWs of 19.7 kDa and 25.6 kDa respectively (Fig 4A and 4B, lane 2). After purification with Ni-NTA column, high purity rSUM in rSUMO group O and rUBC9 were obtained (Fig 4A and 4B, lane 3). PAbs against rSUMO or rUBC9 were obtained from the immunized mice, which could specifically react with the rSUMO or rUBC9 respectively in the lysate of induced *E*.*coli* BL 21 (Fig 4A and 4B, lane 4). The results of western blotting showed that the PAb against rSUMO could react strongly with a 13.5 kDa protein (Fig 4C, Lane 2) in the lysate of *F*. *chinensis* hemocytes, and anti-UBC9 antibodies could react with a 18.7 kDa protein (Fig 4C, Lane 3). No reactive protein bands were observed in control (Fig 4C, Lane 4). The results of IFA showed that PAbs against rSUMO and rUBC9 mainly reacted with the proteins in the nucleus of hemocytes (Fig 4D and 4E), and no green positive signals were observed in control (Fig 4F).

### The effects of silencing of *FcSUMO* and *FcUBC9*

**Fig 5:**
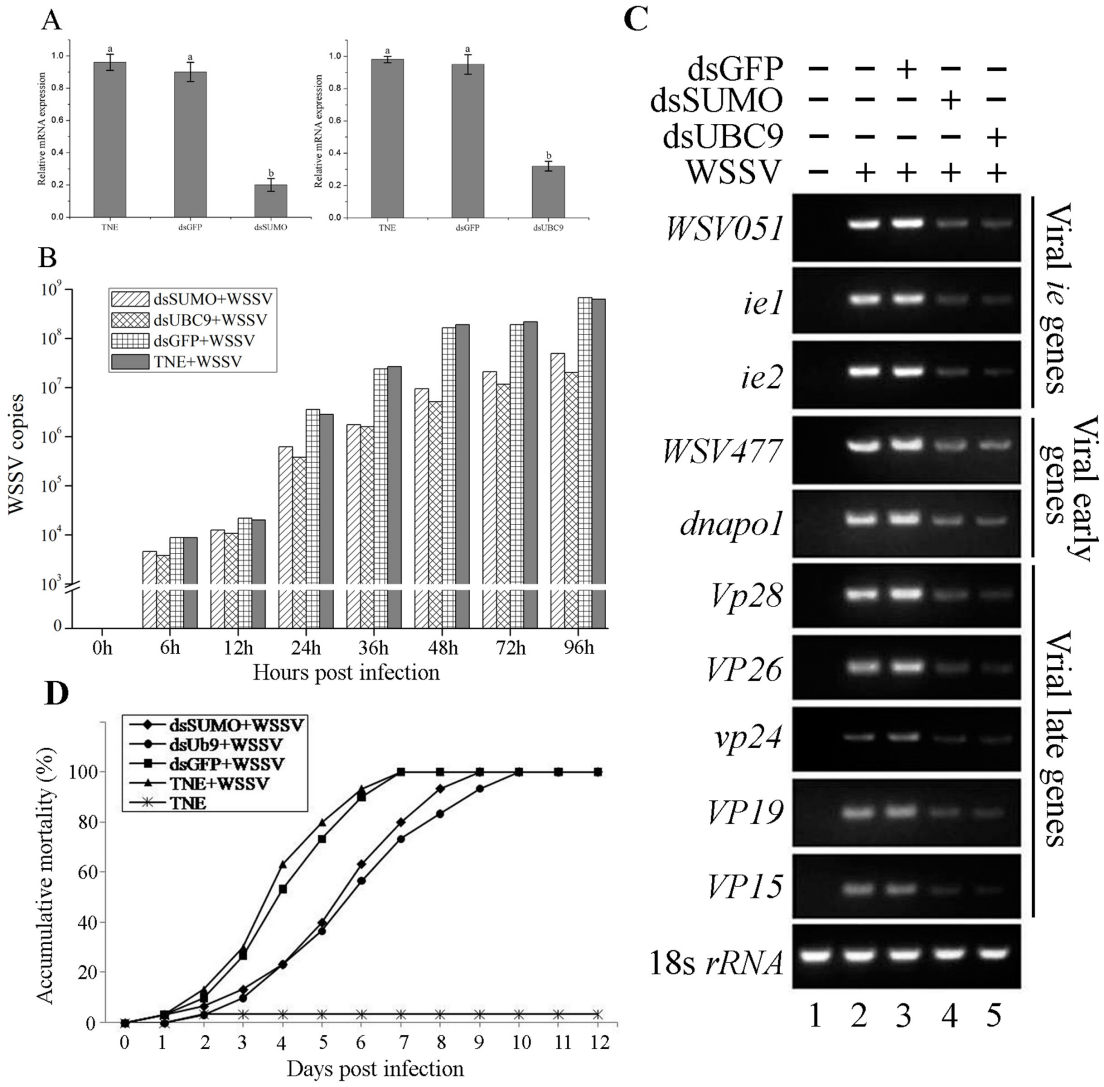
The effects of silencing of *FcSUMO* and *FcUBC9*. **(A)** Silencing efficiencies of dsSUMO and dsUBC9 in hemocytes of *F*. *chinensis* at 48 h post injection of dsRNA. (B) dynamic state of WSSV copies in hemocytes post WSSV infection investigated by real-time PCR; (C) Expression levels of 10 viral genes at 48 hpi, lane 1–4 represent the samples from shrimps injected with TNE+TNE, TNE+WSSV, dsGFP+WSSV, dsSUMO+WSSV and dsUBC9+WSSV respectively; (D) accumulative mortality of shrimp at 1-day interval post WSSV infection.

The *FcSUMO* and *FcUBC9* mRNA expressions were significantly down-regulated at each sampling time after injection of dsRNA (data not shown). The relative expression levels of *FcSUMO* and *FcUBC9* both decreased to their minimum values at 48 h post injection, their silencing efficiencies were 77.8% and 67.7% respectively compared to the dsGFP groups, whereas the expressions of *FcSUMO* and *FcUBC9* were not significantly affected by dsGFP or TNE injection (Fig 5A). At 48 h prior to WSSV challenge, shrimp were injected with dsRNA to characterize the roles of SUMO and UBC9 in regulating viral replication, and the number of WSSV copies in hemocytes samples was calculated. The results showed that the changes of WSSV copies in different groups displayed a similar tendency. The viral copies maintained at a low level at the early stage post infection, and then significantly increased to a high level at 24 hpi, then displayed a stable increase afterward. However, the WSSV copies in dsSUMO and dsUBC9 injection groups were significant lower than that in the dsGFP and TNE injection groups at each sampling time, and the viral number in UBC9 silenced shrimp was a little lower than the number in SUMO silenced shrimp (Fig 5B). In addition, expression levels of ten WSSV genes at 48 hpi were monitored by semi-quantitative RT-PCR. As shown in Fig 5C, the expressions of viral *ie* genes, early genes and late genes were significantly inhibited in SUMO and UBC9 silenced shrimp compared to the control.

To investigate the effects of SUMO and UBC9 knockdown on mortality in WSSV infected shrimp, cumulative mortality of shrimp in each group was calculated. The results showed that the silencing of the two genes both delayed shrimp mortality. Mortality increased steadily post infection and reached to 100% at 9th day in SUMO silenced group and 10th day in UBC9 silenced group. By contrast, in the two positive control groups, 100% cumulative mortality was observed at 7 days post infection. There was almost no mortality in the negative control group (Fig 5D).

## Discussion

In the present work, a SUMO cDNA and a UBC9 cDNA were cloned form hemocytes of *F*. *chinensis* by RACE technique. Multiple sequences alignment of the deduced proteins showed that the two molecules both had significant homology with the ones from various species, and the amino acid sequence of FcSUMO was even completely identical to the SUMOs of *L*. *vannamei* and *P*. *clarkia*, indicating that they are highly evolutionarily conserved. And this finding is consistent with the fact that SUMO and UBC9 polypeptides are conserved from yeast to human [23]. Like the genes identified from other species, *FcSUMO* and *FcUBC9* have their respective active sites, double Gly in SUMO and Cys93 in UBC9, which are crucial in SUMOylation. During SUMOylation, an inactive SUMO is converted to its active form by exposing the C-terminus double Gly residues, which then form a thioester bond with a cysteine of the E1 activating enzyme, subsequently it is transferred onto the active Cys93 of UBC9 and finally passed to the ε-amino group of substrate lysine residues on the target proteins [24]. We speculate that the *FcSUMO* and *FcUBC9* work in a similar way to the SUMOs and UBC9s of other organisms in the SUMOylation process.

Tissue expression profile analysis by qRT-PCR revealed that *FcSUMO* and *FcUBC9* are ubiquitous in the examined tissues, and it was highly expressed in hemocytes and ovary. Similarly, several previous studies have shown the high expressions of SUMO and UBC9 in gonad in other crustaceans, and the function of SUMOylation in testis and ovary developments has been reported [15,16]. However, it was rarely reported that SUMO and UBC9 are highly expressed in hemocytes in other species. In WSSV infection experiment, *FcSUMO* and *FcUBC9* were up-regulated post infection, and the up-regulation extent of these two genes in hemocytes was much higher that in ovary. Similarly, previous study also showed that the mRNA expressions of SUMO and UBC9 were significant up-regulated post WSSV infection in the hepatopancreas and intestine [1]. These results demonstrate that SUMOylation plays an important role in immune response of hemocytes to viral infection.

The localization of *Fc*SUMO and *Fc*UBC9 in hemocytes was analyzed by IFA, and the results showed that the positive signals were mainly observed in nuclei of hemocytes. This result was consistent with the observation in mammals [25–27]. Furthermore, SUMO modification has been implicated in many important cellular processes including the control of genome stability, signal transduction, targeting to and formation of nuclear compartments, cell cycle and meiosis, and these process mainly occurred in nuclear region [28–30]. We speculate that the distributions of the two proteins are essential for their functions in multiple cellular processes.

To date, proteins from many virus families have been shown to be modified by SUMO conjugation, and this modification appears critical for viral protein function. Conversely, viruses can also alter the sumoylation of host proteins to create a cellular environment that facilitates viral survival and reproduction [31]. Gene knockdown using dsRNA has been shown to be a powerful tool for the investigation of gene function in crustacean, which was widely employed to inhibit host genes involved in viral infection in shrimp and displayed a good effect [1, 32–33]. In present research, suppression of SUMO or UBC9 gene transcript levels resulted in the inhibitions of the increase of WSSV copies and the viral gene expressions, and the reduction of shrimp mortality, which indicated that SUMO and UBC9 are involved in WSSV replication. The finding is consistent with the results reported in crayfish [1]. These results suggested that the sumoylation has a close correlation with WSSV infection. However, the specific effects of sumoylation on WSSV remain unclear, so further researches should be performed to lead an in-depth understanding of the relationship between WSSV infection and host sumoylation, which might have some utility for antiviral therapeutics.

## Supporting Information

S1 Fig Full length cDNA sequences and deduced amino acid sequences of *FcSUMO* (A) and *FcUBC9* (B). UBQ domain of SUMO and UBCc domain of UBC9 were shown with underlines, and the active sites (double Gly in SUMO and Cys93 in UBC9) were indicated in ellipses. The polyadenylation signals in the 3’-UTR were also boxed.
(DOC)
