# Supplementary figures and images for: SUMO and SUMO-Conjugating Enzyme E2 UBC9 Are Involved in White Spot Syndrome Virus Infection in Fenneropenaeus chinensis
Source: PLoS One. 2016 Feb 29;11(2):e0150324. doi: 10.1371/journal.pone.0150324 (PMC4771164; doi:10.1371/journal.pone.0150324)

**Figure S1**


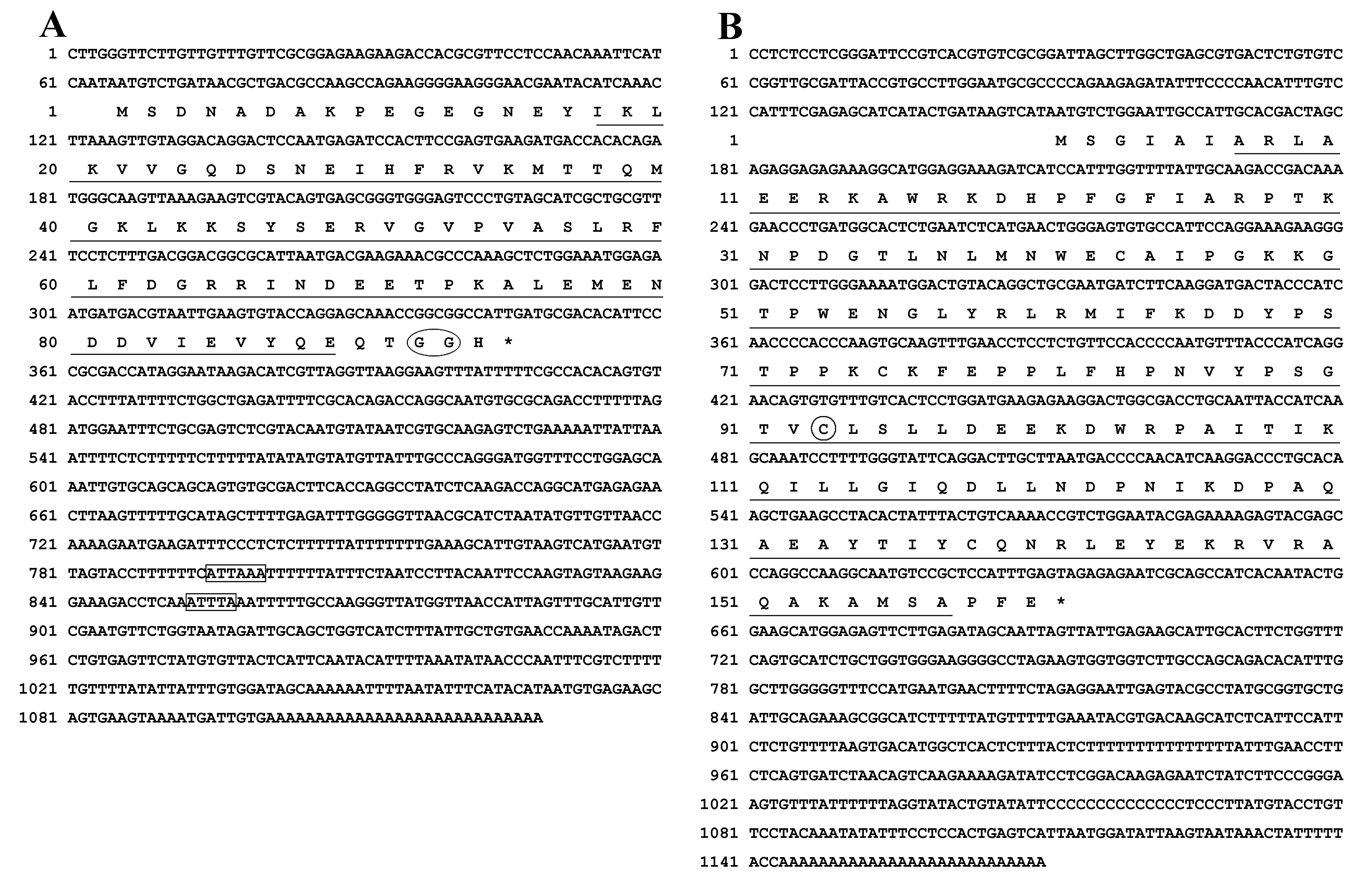

Supplement: S1 Fig — UBQ domain of SUMO and UBCc domain of UBC9 were shown with underlines, and the active sites (double Gly in SUMO and Cys93 in UBC9) were indicated in ellipses. The polyadenylation signals in the 3’-UTR were also boxed. (DOC) [file pone.0150324.s001.doc]
